# Effect of Surface Cleaning Regimen on Glass Ceramic Bond Strength

**DOI:** 10.3390/molecules24030389

**Published:** 2019-01-22

**Authors:** Barbara Lapinska, Jacek Rogowski, Joanna Nowak, Joseph Nissan, Jerzy Sokolowski, Monika Lukomska-Szymanska

**Affiliations:** 1 Department of General Dentistry, Medical University of Lodz, 251 Pomorska St., 92-213 Lodz, Poland; barbara.lapinska@umed.lodz.pl (B.L.); jerzy.sokolowski@umed.lodz.pl (J.S.); 2 Institute of General and Ecological Chemistry, Lodz University of Technology, 116 Zeromskiego St., 90-924 Lodz, Poland; jacek.rogowski@p.lodz.pl; 3 University Laboratory of Materials Research, Medical University of Lodz, 251 Pomorska St., 92-213 Lodz, Poland; joanna.nowak.1@umed.lodz.pl; 4 Department of Oral Rehabilitation, School of Dental Medicine, Tel Aviv University, 6997801 Tel Aviv, Israel; nissandr@post.tau.ac.il

**Keywords:** ceramics, surface cleaning methods, saliva contamination, TOF-SIMS, mass spectrometry, leucite ceramics, lithium disilicate ceramics, bond strength

## Abstract

This study investigated the effect of saliva contamination on chemical changes of ceramic surface as well as the influence of saliva cleaning methods on ceramic-resin bond strength. Saliva was used to contaminate leucite (LGC) and lithium disilicate (LDGC) glass ceramic surfaces. The following cleaning methods were tested: water spray, cleaning with orthophosphoric acid, universal cleaning paste, ultrasonic cleaning with water, re-etching with hydrofluoric acid. Non-contaminated ceramic sample served as control. Chemical analysis of ceramic surfaces was performed using time-of-flight secondary ion mass spectrometry (TOF-SIMS). Shear bond strength (SBS) of ceramics to resin material was tested after 24-hour water storage and after thermocycling. The most effective cleaning method of saliva-contaminated ceramic surface was cleaning LGC surface with orthophosphoric acid or re-etching the LDGC surface with hydrofluoric acid. The application of the following methods resulted in obtaining reliable bond strength.

## 1. Introduction

Glass ceramic resin bonded restorations gained wide recognition due to their outstanding aesthetics and clinical durability. Luting all-ceramic restorations to tooth structure is one of the crucial steps influencing their clinical performance. The ceramic surface requires proper adhesive treatment in order to achieve micromechanical retentive surface structure, as well as to enhance chemical bonding to resin cement via silane application [1,2]. For adhesive cementation of glass ceramic restorations, ceramic surface treatment involves air-borne particle abrasion and etching with hydrofluoric acid (HF) [1,3,4,5,6,7]. Usually, such a treatment takes place in a dental laboratory, resulting in delivery of pre-etched ceramic restoration to the dental office. Hydrofluoric acid-etching of silica-based glass ceramics produces a high-energy surface, which is highly retentive, but also easily contaminable [8]. Unfortunately, during clinical try-in procedures, the ceramic surface usually becomes contaminated with try-in paste, blood, or saliva.

Saliva is a mixture of water, white blood cells, epithelial cells, glycoproteins, enzymes, immunoglobulins, enzymes, mucins, and nitrogenous products, such as urea and ammonia. Various electrolytes, including sodium, potassium, calcium, magnesium, bicarbonate, and phosphates are among other saliva components [9]. It also contains bacteria and food debris. Such organic debris remnants on the ceramic surface might adversely affect ceramic-resin material bonding effectiveness [10,11,12,13,14]. On saliva contamination, salivary proteins adhere to the surface of dental tissues, dental materials, or restorations forming an acquired pellicle, changing the wettability and surface free energy of the substrate [15].

After saliva contamination of leucite and lithium disilicate glass ceramic surface, significant decrease in total surface free energy (SFE) was reported, which might eventually have a detrimental effect on the bonding capability of the ceramic surface to dental adhesive and resin materials [13,14]. However, the ceramic surface is the least prone to pellicle formation and plaque accumulation among all dental restorative materials [16,17]; acquired pellicles enhance bacteria adhesion leading to the degradation of adhesive bonding and secondary caries.

Therefore, in order to overcome these potential clinical problems, the ceramic surface should be cleaned from any contaminants prior to adhesive cementation. It was shown that saliva contamination could not be removed with water rinsing [11]. Various cleaning methods of the saliva-contaminated ceramic surface were recommended, including cleaning with tap or distilled water, 0.5% or 5% sodium hypochlorite solution, 2% chlorhexidine, 96% ethanol, 70% isopropanol, ultrasonic cleaning, phosphoric (H_3_PO_4_) or hydrofluoric (HF) acid etching, or cleaning pastes [10,11,12,14,18,19,20,21,22,23]. Other researchers brought up the importance of silane reapplication in obtaining reliable bond strength after ceramic surface cleaning [10,11,24].

Mass spectrometry is often used in the study of dental materials including ion release [25,26,27,28,29]. In recent years, time-of-flight secondary ion mass spectrometry (TOF-SIMS) has been increasingly used in the research of dental materials [30]. The TOF-SIMS allows for chemical qualitative surface analysis of samples ranging from biological tissues to electronic devices [31,32]. The method was applied by some authors investigating surface coatings, ceramic surface contamination [1], regenerative mineralization of biomedical implants, or to examine the zirconium phosphate (ZrP) compound [26,33].

The aim of the study was to investigate the effect of various surface cleaning regimens of saliva-contaminated glass ceramic surfaces on the surface chemical composition and shear bond strength to resin material.

## 2. Results

### 2.1. Surface Elemental Analysis

**Figure 1:**
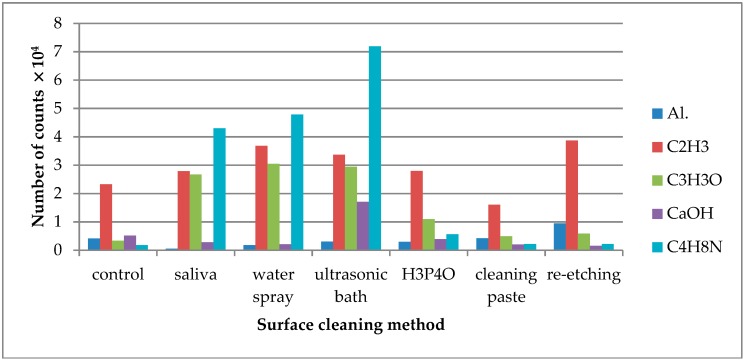
Number of counts of positive ions emitted from the surface of leucite glass ceramic (LGC) after various surface cleaning methods.

**Figure 2:**
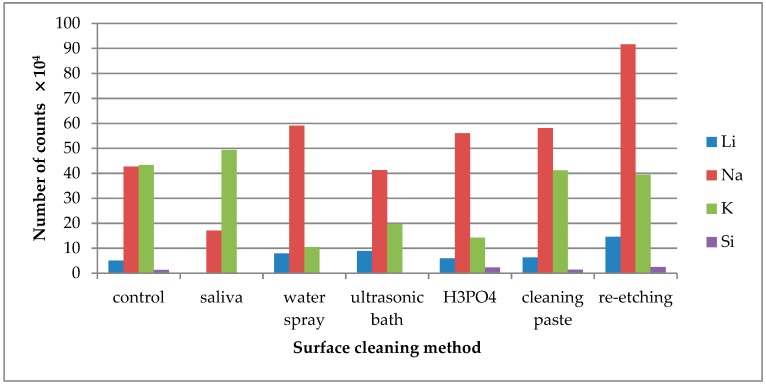
Number of counts of positive ions emitted from the surface of leucite glass ceramic (LGC) after various surface cleaning methods.

**Figure 3:**
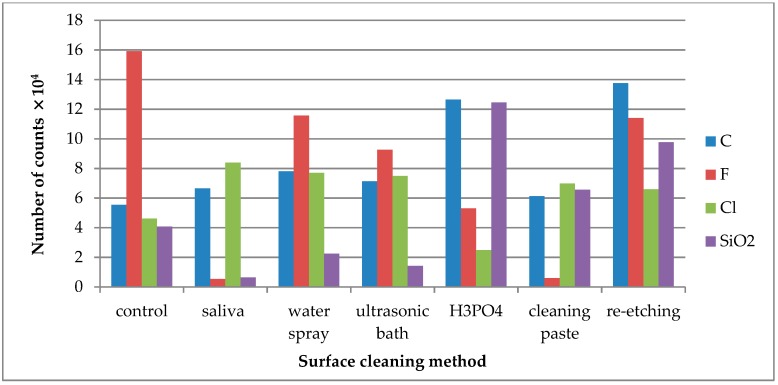
Number of counts of negative ions emitted from the surface of leucite glass ceramic (LGC) after various surface cleaning methods.

TOF-SIMS results for leucite ceramic (Avanté® Micro Crystal Porcelain) surface are presented in Figure 1, Figure 2 and Figure 3. Since the emission intensities of Li^+^, Na^+^, K^+^, and Si^+^ ions were much higher than those of the other positive ions, they are presented in separate figures.

Saliva contamination of the LGC surface resulted in higher (when compared to the control group) emission of C_2_H_3_^+^, C_3_H_3_O^+^, and C_4_H_8_N^+^ ions, which supposedly derived from saliva organic compounds. It is noteworthy that C_2_H_3_^+^ ions showed significant emission from the reference sample, which indicated that they were also characteristic for the components of the ceramics. However, it should be assumed that the C_2_H_3_^+^ ions were generated from the saliva components covering the surface of the sample as well. It was confirmed by the moderate increase in the emission of C_2_H_3_^+^ ions observed from the contaminated sample. If these ions originated only from the ceramics, the saliva on the surface of the sample should cause a decrease in their emission intensity. However, due to the fact that C_2_H_3_^+^ ions originated both from ceramics and saliva components, they could not be used to assess the effectiveness of ceramic surface cleaning methods. Also higher emissions of K^+^, C^−^, and Cl^−^ ions that come from saliva [34] were detected. On the other hand, lower emission of F^−^ and SiO_2_^−^ ions that are components of ceramic materials were observed, suggesting the saliva coverage of the ceramic surface.

The highest emission of Al^+^ ions was observed, when re-etching of saliva-contaminated LGC surface was performed (Figure 1). As for C_3_H_3_O^+^, C_4_H_8_N^+^ ions, characteristic for saliva organic compounds, the TOF-SIMS results showed that they were most effectively removed from saliva-contaminated LGC surface using universal cleaning paste. The other tested cleaning methods, such as water spray or ultrasonic cleaning, showed the least effectiveness. Other ions indicating effectiveness of the saliva cleaning methods were Ca^+^ ions. Their emission level decreased after saliva contamination of the LGC surface. After cleaning the contaminated surface with H_3_PO_4_, the Ca^+^ ion emissions rose, indicating cleaning effectiveness of the method. The slight increase in Ca^+^ ion emissions from the contaminated surface cleaned with ultrasonic bath might result from rinsing the ions from inside of the specimens on the surface induced by ultrasounds.

Saliva contamination of the LGC surface significantly decreased Li^+^ and Na^+^ ion emission intensity. The highest emission of these ions was noted after re-etching saliva-contaminated LGC surface (Figure 2).

The increase in K^+^ ion (present in saliva) emission was observed from saliva-contaminated LGC surface. The lowest intensity of K^+^ emission was observed after cleaning the surface with water spray, H_3_PO_4_, or ultrasonic cleaning. As for Si^+^ ions, the emission intensity detected in TOF-SIMS was too low for the credible analysis.

C^−^ ions were detected for all tested LGC specimens as C^−^ derives from all kinds of contaminants, quickly adsorbing on the specimens’ surface, including the cleaned one (Figure 3). Cl^−^ ions were present on the control specimen of LGC. After contaminating the specimen’s surface with saliva, the emission of Cl^−^ ions increased and was stable after performing all tested surface cleaning methods, except cleaning with H_3_PO_4_. F^−^ is one of the ceramics’ components, as its emission dropped substantially after saliva contamination of the LGC surface. High levels of F^−^ ion emissions from the saliva-contaminated LGC surface cleaned with water spray or re-etching method was observed. The highest decrease in F^−^ ion emissions was observed after using cleaning paste on the contaminated surface. SiO_2_^−^ is another ion present on the control specimen’s surface. The large increase in SiO_2_^−^ emission after HF etching may suggest that it is one of the ceramics’ components.

**Figure 4:**
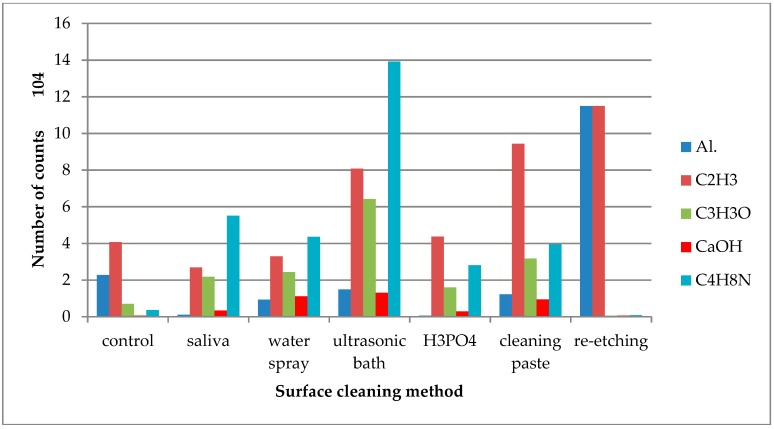
Number of counts of positive ions emitted from the surface of lithium disilicate glass ceramic (LDGC) after various surface cleaning methods.

**Figure 5:**
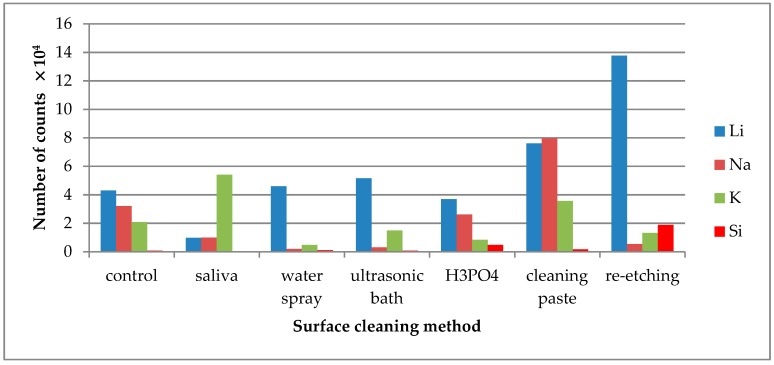
Number of counts of positive ions emitted from the surface of lithium disilicate glass ceramic (LDGC) after various surface cleaning methods.

**Figure 6:**
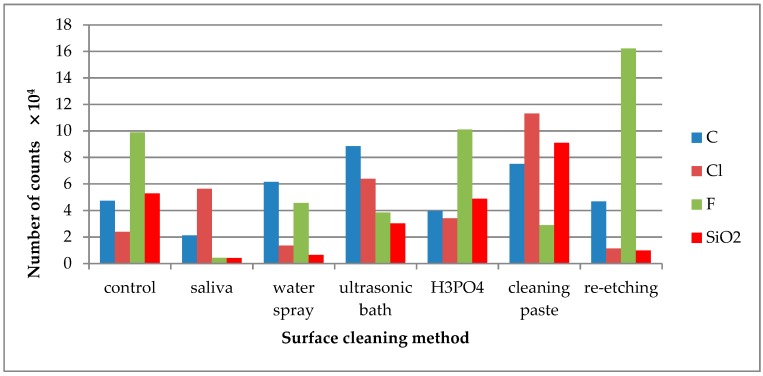
Number of counts of negative ions emitted from the surface of lithium disilicate glass ceramic (LDGC) surface after various surface cleaning methods.

TOF-SIMS study results for lithium disilicate ceramics (IPS e.max) were presented in Figure 4, Figure 5 and Figure 6.

On the saliva-contaminated LDGC surface, the increased emission of C_3_H_3_O^+^ and C_4_H_8_N^+^ ions was observed when compared to the control. Based on the observations, these ions were identified as derived from saliva organic compounds. Also, higher emissions of K^+^ and Cl^−^ ions from saliva components were detected. Saliva contamination of LDGC surface caused the decrease in emission of Al^+^ ions, derived from ceramics’ components, when compared to the control. The highest emission of Al^+^ ions was observed for re-etching of saliva-contaminated LDGC surface (Figure 4). Re-etching the LDGC surface with HF also resulted in a significant drop in emission of C_3_H_3_O^+^, C_4_H_8_N^+^, and CaOH^+^ ions.

Li^+^ ions are one of the ceramics’ constituents and their highest emission intensity was observed from the saliva-contaminated LDGC specimen cleaned with re-etching method (Figure 5). Na^+^ ions emission intensity significantly dropped when water spray, ultrasonic cleaning, or re-etching was applied. The emission intensity of K^+^ ions increased after saliva contamination and dropped significantly after application of all tested cleaning methods, except the use of cleaning paste. As for Si^+^ ions, the emission intensity detected in TOF-SIMS was too low for credible analysis. However, the highest emission of Si^+^ ions was observed after re-etching.

The emission of C^−^ ions was detected in all tested specimens (Figure 6). C^−^ ions might come from carbon-doped contaminations that quickly adsorbed on the surface after the cleaning process. The saliva derived Cl^−^ ions were effectively removed after cleaning with water spray or re-etching. F^−^ was one of the ceramic components. The high emission of F^−^ ions from the saliva-contaminated LDGC surface cleaned with re-etching might prove the high cleaning effectiveness of that method. SiO_2_^−^ ions are the components of the LDGC and were already present on the control specimen. Saliva contamination caused the decrease in emission intensity of these ions. The effective cleaning (the increase in SiO_2_^−^ ions emission) was observed when cleaning paste or cleaning with H_3_PO_4_ was performed.

### 2.2. Bond Strength

SBS results for LGC after performing different cleaning regimens of saliva contaminants and for the control group were presented in Table 1.

For the general model used (Kruskal-Wallis test) the differences in SBS values obtained in study groups after 24-hour storage were considered statistically insignificant (*p* > 0.05). Mean SBS values obtained for study groups using H_3_PO_4_, cleaning paste or ultrasonic bath as surface cleaning methods were comparable (~12 MPa). Pairwise comparison showed that re-etching resulted in significantly lower SBS (*p* < 0.01) than cleaning with water spray (*p* = 0.0073). Also, the difference in SBS produced by water spray cleaning and cleaning paste was close to significance level (*p* = 0.054).

**Figure 7:**
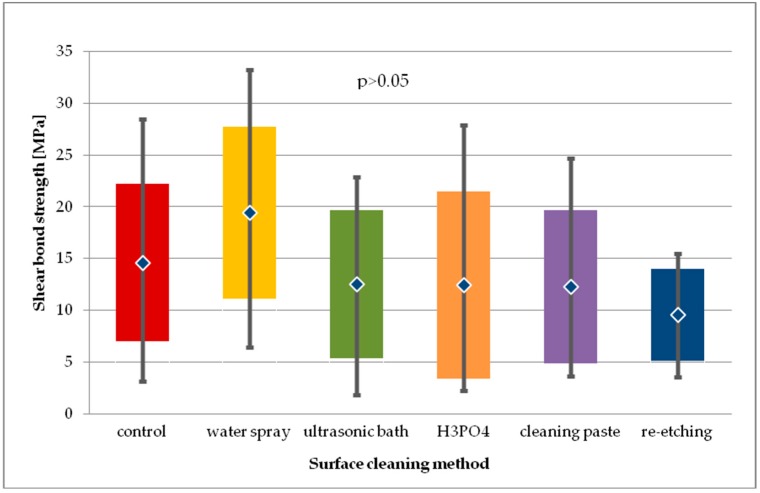
Shear bond strength of resin material to saliva contaminated leucite glass ceramics (LGC) using different cleaning methods, after 24-hour water storage.

When comparing the SBS of study groups to control group after 24-hour storage, no statistical difference was observed (*p* > 0.05) (Figure 7).

**Figure 8:**
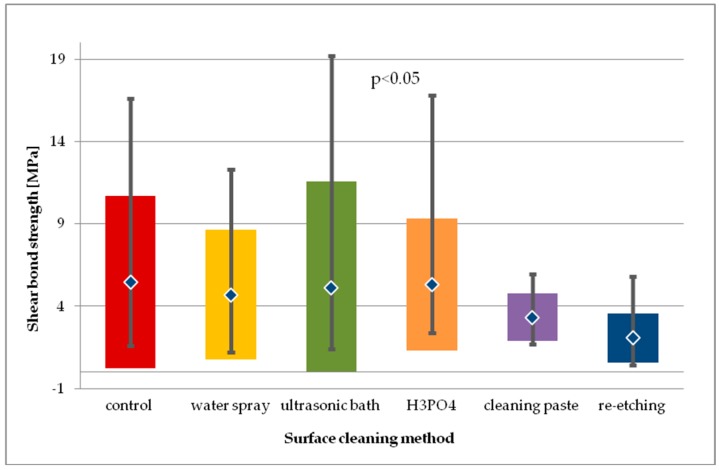
Shear bond strength of resin material to saliva contaminated leucite glass ceramics (LGC) using different cleaning methods, after 24-hour water storage followed by thermocycling.

Within the batch of LGC specimens subjected to thermocycling (Figure 8), the highest SBS values were observed in study groups using H_3_PO_4_ and ultrasonic cleaning. For general model used, statistically significant differences in SBS values were observed (*p* < 0.05). Pairwise comparison was performed and three statistically significant differences in SBS values were observed. Cleaning with water spray, H_3_PO_4_, and cleaning paste resulted in statistically significantly higher SBS values than those obtained after re-etching with 9% HF for 20 s (*p* < 0.05). Other pairwise comparisons of study groups showed no statistical difference in SBS. Also, no statistical difference in SBS of study groups was observed in comparison to control group (*p* > 0.05).

When comparing SBS results for each study group after 24-hour storage and after thermocycling, a statistically significant decrease in SBS values was observed for the following cleaning methods: water spray (*p* < 0.01), cleaning paste (*p* < 0.01), re-etching (*p* < 0.001), ultrasonic cleaning (*p* < 0.05). The greatest decrease in SBS after thermocycling was reported for the group using re-etching saliva-contaminated LGC surface with HF as cleaning method.

SBS results for LDGC after performing different cleaning regimens of saliva contaminants and for the control group were presented in Table 2.

**Figure 9:**
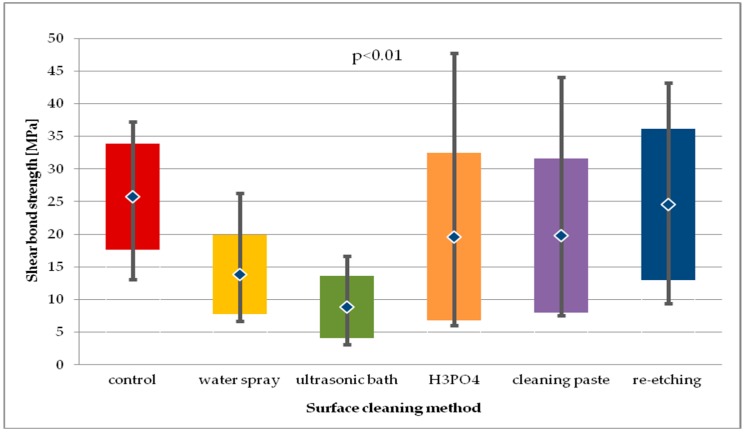
Shear bond strength of resin material to saliva contaminated lithium disilicate glass ceramics (LDGC) using different cleaning methods, after 24-hour water storage.

In case of LDGC, the highest SBS values after 24-hour storage were recorded in study group, where re-etching was used. The results were comparable to the one of control group. Using H_3_PO_4_ as well as cleaning paste showed similar SBS values. The lowest SBS values were obtained in the group with water spray cleaning. For the general model used, the differences in SBS values of LDGC obtained in study groups after 24-hour storage were considered statistically significant (*p* < 0.01) (Figure 9). Pairwise comparisons revealed that SBS values for re-etching were significantly higher than for cleaning with water spray (*p* < 0.01). Also, ultrasonic cleaning was found to result in lower SBS values than water spray (*p* < 0.05), H_3_PO_4_ cleaning (*p* < 0.01), using cleaning paste (*p* < 0.05), or re-etching (*p* < 0.001).

When compared to control group, water spray or ultrasonic cleaning showed significantly lower SBS (*p* < 0.01 and *p* < 0.001, respectively). It is worth noticing that in other study groups the SBS values were also lower than in the control group, however the differences were statistically insignificant.

**Figure 10:**
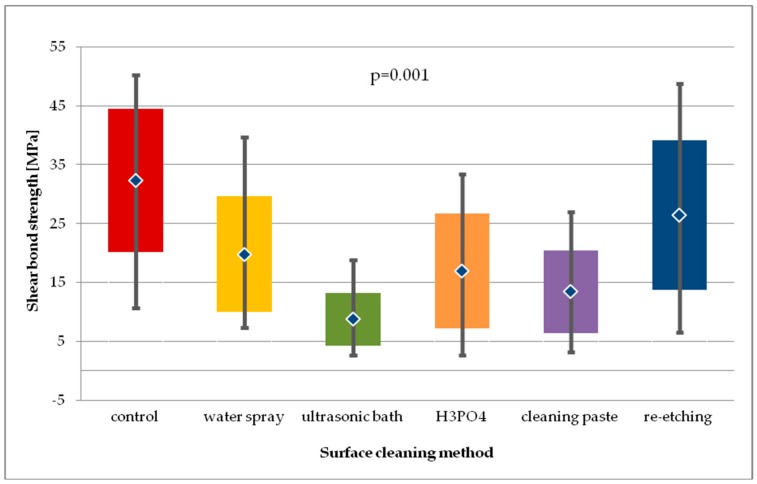
Shear bond strength of resin material to saliva contaminated lithium disilicate glass ceramics (LDGC) using different cleaning methods, after 24-hour water storage followed by thermocycling.

After thermocycling of LDGC specimens (Figure 10), still the highest SBS values were recorded for re-etching and for the control group, while the lowest were for the group where cleaning paste was applied. For the general model used, differences in SBS values were close to statistical significance (*p* = 0.055). Pairwise comparison revealed that SBS values for re-etching group were significantly higher than of the cleaning paste group (*p* < 0.01).

The differences in SBS values for each study group of LDGC specimens after 24-hour storage followed by thermocycling was found to be statistically significant (*p* = 0.001). Pairwise comparison showed that ultrasonic cleaning resulted in significant lower SBS values than water spray cleaning (*p* < 0.01), H_3_PO_4_ cleaning (*p* < 0.05), or re-etching (*p* < 0.001). Also, re-etching was found to produce higher SBS than using cleaning paste (*p* < 0.01). However, SBS values obtained after thermocycling in all study groups, except for re-etching, were significantly lower than in the control group.

Statistical analysis of SBS values obtained after 24-hour storage and after thermocycling for all study groups showed no significant differences (*p* > 0.05). However, SBS values of water spray and re-etching group were the ones that did not decrease when subjected to thermocycling.

## 3. Discussion

The present study investigated the influence of different saliva cleaning regimens of a hydrofluoric acid etched glass ceramic surface on bond strength to resin material, as well as the effect of artificial ageing on bond strength. The study used the TOF-SIMS technique, involving a highly sensitive secondary ion mass spectrometer, allowing for detection of elemental and molecular ions, delivering information from the upper layer of the investigated material [35]. The method was previously used by authors, aside from Energy Dispersive Spectroscopy (EDS), to evaluate changes in chemical composition on glass ceramic surfaces due to applied surface treatment [1,20]. The present study observed that the elemental composition of superficial part of the ceramic surface evolved, while different cleaning methods were introduced.

Followed by saliva contamination of the ceramic surface, non-covalent adsorption of salivary protein occurs, resulting in creation of an organic coating, impossible to remove by rinsing with tap water [22]. That thin organic film, observed in SEM, covering the hydroxyl sites of the ceramic surface may be responsible for its reduced potential for hydrophilic interactions (lower polarity and hydrogen-bonding forces) [14]. The main cations present in saliva are Ca^2+^, Mg^2+^, Na^+^, K^+^. Among anions, H_2_PO_4_^−^, HPO_4_^2−^, PO_4_^3−^, HCO_3_^−^, CO_3_^2−^ Cl^−^, F, and SCN^−^ are recognized. Since saliva also contains various organic compounds, other ions might also be detected using TOF-SIMS method. In the present study, C_3_H_3_O^+^, C_4_H_8_N^+^, K^+^, C^−^, and Cl^−^ ions were identified as saliva contaminants’ constituents. Ions, such as C_3_H_3_O^+^ and C_4_H_8_N^+^, are simple ions that derive from fragmentation of saliva organic compounds. These compounds (peptides, amines, glycoproteins) contain C and H, and also N and O [36,37]. Assigning C_3_H_3_O^+^ and C_4_H_8_N^+^ ions as constituents of saliva organic compounds was confirmed by the fact that their emission intensity, detected with TOF-SIMS technique, significantly rose after contaminating the ceramic surface with saliva. Thus, saliva seemed to be their only possible source. The high emission of these ions from a saliva-contaminated, non-cleaned ceramic surface was observed, whereas no emission of PO_3_^−^ and/or CO_3_^−^ ions was detected.

Saliva contamination of a ceramic surface results in altering the surface wettability and possibly decreasing bond strength to resin material. Upon adhesive cementation, contaminants should be removed in order to obtain the durable adhesion and clinical performance of the ceramic restoration [11].

Hydrofluoric acid etching of glass ceramic produces a porous surface by dissolving and removing the surface layer of the glassy matrix containing silica (SiO_2_), silicates (SiO_4_^4−^), and leucite crystals (K_2_O•Al_2_O_3_•4SiO_2_) [38,39,40]. In the present study, the high emission of Al^+^, Li+, and Na+ ions from the re-etched surface of saliva-contaminated LGC was observed. It might prove the very good cleaning effectiveness of the method, as these ions are derived from components of the ceramic surface (Table 3). However, it might also indicate that the additional HF etching of the ceramic surface excessively dissolved the ceramic glassy phase composed mainly of silica, exposing the aluminous crystalline substrate, hence the high emission of Al^+^ ions. The re-etching of the saliva-contaminated LGC surface also resulted in a high level of F^−^ ion emission, suggesting high effectiveness of the cleaning method or presence of fluoride “debris” left after HF etching [41]. The lowest emission of fluoride ions was detected after using cleaning paste, indicating its low cleaning effectiveness.

Considering the effectiveness of the tested cleaning methods in terms of ceramic-resin bond strength, the differences in SBS values between study groups were statistically insignificant. Yet the re-etching with HF of saliva-contaminated LGC resulted in substantially lower SBS than water spray cleaning and dropped significantly after thermocycling. Only using H_3_PO_4_ cleaning produced durable bonding, which did not significantly deteriorate after thermocycling. The following results are consistent with the findings of Aboush et al. [11], reporting that the use of H_3_PO_4_ to clean the saliva-contaminated ceramic surface was the most beneficial. According to Yoshida et al. [14], both methods—etching with hydrofluoric and cleaning with H_3_PO_4_—were effective in removing contaminants from ceramic surfaces as well as in creating an adhesive surface for bonding. The poor SBS results for re-etching method obtained in the present study might be explained by the weakening effect of prolonged HF etching on the LGC surface microstructure, causing the collapse of highly extended ceramic surface area or its fracture [42,43,44]. Cleaning with H_3_PO_4_, using cleaning paste or ultrasonic cleaning, allowed for obtaining comparable bond strength to non-contaminated (with saliva) LGC surface.

It was reported that silanization prior to saliva contamination protected the surface of the glass ceramic and resulted in higher bond strengths [11]. Yet, Nikolaus et al. [10] investigated that cleaning a saliva-contaminated, previously silanized ceramic surface with water spray is not sufficient. They achieved the best ceramic-resin bond strength results when cleaning of the surface with water or ethanol was followed by application of another silane layer.

Analyzing the effectiveness of saliva contamination cleaning methods used on the LDGC surface, the re-etching with HF method was found to be the most successful one. The assumption was made upon observing (in TOF-SIMS study) the highest emission of ions derived from the ceramics’ composition, i.e., Al^+^, Li^+^, F^−^, after using the abovementioned method. High emission of Li^+^ ions might derive from excessive dissolution of lithium disilicate crystals, whereas F^−^ ions might also indicate the presence of debris (fluoride residues) left on the ceramic surface after HF etching. Also, the emission of saliva ions, i.e., C_3_H_3_O^+^, C_4_H_8_N^+^, CaOH^+^, K^+^, and SiO_2_^−^, dropped significantly after the re-etching. Klosa et. al. [12] reported the re-etching of LDGC surface with HF acid to be the most effective in removing saliva contamination, also when silicone disclosing medium was used. However, hydrofluoric acid is proven to be a caustic compound, being both volatile and toxic, thus presenting a health hazard [45]. Cleaning with H_3_PO_4_ is one of the techniques proved to be effective in removing saliva contaminants from the silica-based ceramic surface after try-in procedure [12]. In the present study, using H_3_PO_4_ allowed for achieving comparable SBS values to cleaning paste, but for the former, the bond strength did not deteriorate after ageing.

In the case of LDGC, the re-etching of the saliva-contaminated surface was found to be the most effective cleaning method. The method resulted in a ceramic-resin bond strength almost as high as for non-contaminated ceramic surface. Thermocycling did not significantly influence the bond strength in the study groups, however the greatest decrease in SBS value was observed for the study group, where cleaning paste was used. 

A commercially available cleaning paste product designed for ceramic surface decontamination (Ivoclean/Ivoclar Vivadent) was used in the study [46]. The cleaning paste is composed of an alkaline suspension of zirconium oxide particles that preferentially bind the phosphate contaminants from saliva (phosphoprotein), resulting in a clean ceramic surface. In the present study, using cleaning paste on the saliva-contaminated surface of LDGC did not allow to achieve SBS values comparable to the control group. A previous study mentioned HF etching or using cleaning paste as being effective in cleaning saliva-contaminated LDGC surface [47].

Takahasi et al. [19] investigated the use of various cleaning methods of saliva-contaminated zirconia ceramic surface, e.g., cleaning with water, universal cleaning paste (Ivoclean), or paste containing phosphoric acid monomer (Multi Etchant). Authors reported the use of commercial cleaning agents to be the most effective. Alnassar et. al. [47] reported using 96% isopropanol, 34% orthophosphoric acid, 5% HF acid, or Ivoclean to remove saliva mixed with silicone disclosing medium from LDGC surface. Application of these methods resulted in lower bond strength, when compared to uncontaminated ceramics. The other study, however, indicated that the most effective and determining durable bond strength was cleaning the saliva-contaminated LDGC surface with the Ivoclean paste [48].

Despite the differences in surface chemical composition observed in TOF-SIMS study, they could not be directly translated into SBS results obtained for tested ceramics. Other studies reported that no major differences were observed in surface topography of ceramics after application of surface cleaning methods [12,20,48]. The ceramic-resin bond strength depends on many factors, among which micromechanical retention plays a significant role. The tested LGC contains much smaller and rounder leucite crystals compared to conventional porcelain. After firing, the leucite crystals are suspended in a three dimensional network within a glass matrix. The ceramics’ microstructure resembles the honeycomb-like structure of human enamel. After application of H_3_PO_4_ on HF etched, saliva-contaminated LGC surface, the highest emission of SiO_2_^−^ ions was observed. It might indicate a substantial amount of disclosed leucite crystals on the ceramic surface, creating a retentive microstructure.

On the other hand, LDGC microstructure consists of small, interlocking, densely packed, needle-like lithium disilicate crystals that are randomly oriented, with the addition of much smaller secondary lithium orthophosphate crystals. That itself provides greater micromechanical retention of the surface influencing the potential bond strength. In the present study, the SBS values noted for LDGC were much higher than for LGC. After re-etching of the HF etched, saliva-contaminated LDGC surface, high emission of Li^+^ and Si^+^ ions was noted. It might indicate the greater amount of exposed lithium disilicate crystals on the surface compared to control group, yielding the highest SBS results observed for that cleaning method. For both tested ceramics, the cleaning method yielding the highest SBS results also presented the lowest emission of C_4_H_8_N^+^ ions in TOF-SIMS study. The C_4_H_8_N^+^ ions are components of saliva; therefore, their low emission from the saliva-contaminated ceramic surface might indicate the effectiveness of the cleaning method.

The study used flowable composite instead of resin cement as the major interests of the study were changes in the glass ceramic surface and their influence on the bond strength to resin material. Flowable composite resin and composite resin cement possess comparable physicochemical properties due to similar polymer matrix and similar filler content. Hence, the cementation techniques using composite resin were introduced in clinical practice. Also, based on the previous studies [3,4], the presence of cohesive and adhesive failures equally distributed among study groups legitimizes the use of a flowable composite in the bond strength study design.

In the present study, the most effective (and yielding highest SBS) cleaning methods of the HF etched, saliva-contaminated surface of LGC and LDGC were H_3_PO_4_ application and HF re-etching, respectively. These results were in agreement with Yoshida et. al. [14], who reported that cleaning methods using HF and H_3_PO_4_ improved bond strength of saliva-contaminated LGC and LDGC surfaces.

## 4. Materials and Methods

**Figure 11:**
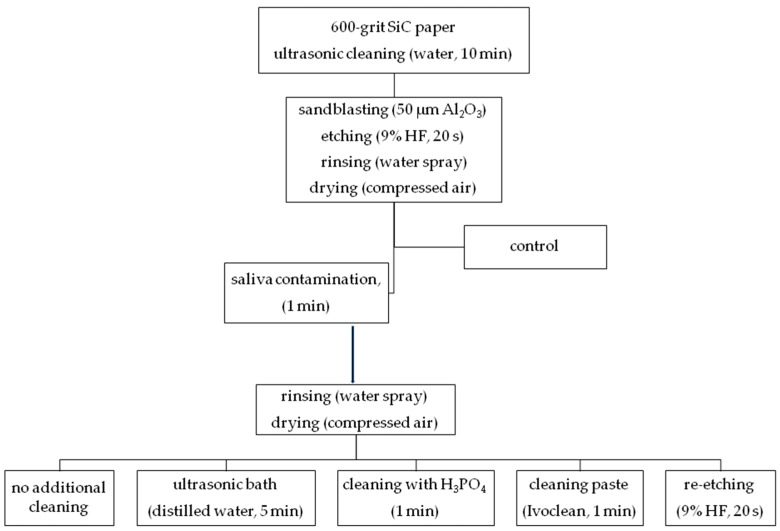
Ceramic surface cleaning methods used in the study (study design).

Disk-shaped samples (4 mm in height, 5 mm in diameter) of two commercially available glass ceramics—microcrystal leucite, alumino-silicate glass ceramics (LGC) (Avanté Micro Crystal/Pentron) and lithium disilicate glass ceramics (LDGC) (IPS e.max Press/Ivoclar Vivadent)—were fabricated according to manufacturers’ instructions. Samples were invested in auto-polymerized poly(methyl methacrylate) (PMMA) resin (Villacryl IT, Zhermack Dental, Italy) in polyvinyl chloride (PVC) rings, followed by placing in tap water in order to reduce the temperature rise due to the exothermic polymerization reaction of acrylic resin. Surfaces of ceramic samples were wet ground with 600-grit SiC papers in order to obtain a flat surface. Afterwards, specimens were ultrasonically cleaned with water for 10 min (using EasyClean/Renfert). The surface was sandblasted with 50 µm Al_2_O_3_, with pressure of 3.5 bars at a 45-degree angle from 15 cm distance. Then, the surface was etched with 9% HF (Ultradent Porcelain Etch/Ultradent) for 20 s, rinsed with water spray for approximately 1 minute, and dried with compressed air. Samples were immersed in saliva (0.5 mL for each sample) for 1 min at 37 °C. Saliva was collected from one healthy female donor (main researcher), who restrained from eating and drinking for 2 h prior to saliva donation for the experiment. All samples used fresh saliva, collected at the same occasion. Samples were randomly divided into groups (n = 23) and different cleaning regimens of saliva-contaminated ceramic surface were performed (Figure 11). The surface of group 1 samples was rinsed with water spray only. In group 2, specimens were ultrasonically cleaned in distilled water for 5 min (using EasyClean/Renfert) and then air-dried. Group 3 used additional cleaning with 34% orthophosphoric acid (H_3_PO_4_) applied on the ceramic surface for 1 minute, then water-sprayed and air-dried. Group 4 used water spray followed by universal cleaning paste (Ivoclean) applied on the surface and rubbed into it for 20 seconds, then water-sprayed and air-dried. Group 5 used additional etching of the surface with 9% HF for 20 s (re-etching), then water-sprayed and air-dried. The specimens without saliva contamination served as the control group (group 6). All specimens were prepared by one operator.

All the materials used in the study were presented in Table 3.

### 4.1. Surface Elemental Analysis

The time-of-flight secondary ion mass spectrometry with a TOF-SIMS IV mass spectrometer (ION-TOF GmbH, Muenster, Germany) was used to study changes in elemental composition of the outmost monolayer of ceramic surface after performing different cleaning regimens. The instrument was equipped with Bi^+^ liquid metal ion gun and high mass resolution time of flight mass analyzer. Secondary ion mass spectra were recorded from an approximately 100 × 100 µm^2^ area of the sample surface. During measurement, the analyzed area was irradiated with the pulses of 25 keV Bi^3+^ ions at 10 kHz repetition rate and an average ion current 0.4 pA. The secondary ions emitted from the bombarded surface of the sample were separated and analyzed using a time-of-flight analyzer. The analysis time was 30 s, giving an ion dose below the static limit of 1 × 10^13^ ions/cm^2^. One specimen of each ceramics from each study group was subjected to the examination (12 specimens in total). In order to identify ions distinctive for saliva contamination (saliva constituents), an additional specimen of each ceramics was prepared to be used as a reference sample. The surface treatment was alike in other study groups, followed by immersion in saliva, but without performing any cleaning procedure.

Based on the SEM-EDS analysis of ceramic surface composition performed in the previous studies [1,20], the following ions emitted from the ceramic surface were chosen for the analysis: Li^+^, Na^+^, K^+^, Si^+^, Al^+^, C_2_H_3_^+^, C_3_H_3_O^+^, CaOH^+^, C_4_H_8_N^+^, C^−^, F^−^, Cl^−^, SiO_2_^−^. The number of ion counts for individual samples was normalized to the number of counts of all ions emitted from the reference sample. This means that the number of ions emitted from the surface of each sample was multiplied by the ratio of the total number of counts of ions from the reference sample to the sample analyzed.

### 4.2. Bond Strength

In order to perform bond strength testing, universal primer (Monobond Plus/Ivoclar Vivadent) was applied on the ceramics’ surface, left for 60 s to react, and then the remaining excess of it was dispersed with a strong stream of air. Next, bonding agent (XP Bond/Dentsply) was applied according manufacturer’s instructions. Afterwards, 1-mm thick layer of flowable composite (X-flow/Dentsply) was applied using a silicone ring (4 mm of height, 3 mm in diameter) and polymerized with an LED polymerizing lamp (Demetron A.2, Kerr, Switzerland), followed by incremental application and polymerization of condensable composite (Spectrum TPH®3/Dentsply) [3,4,21,49].

All samples (6 groups for each ceramics, n = 22, 264 samples in total), were stored in distilled water for 24 h at 37 °C. Next, half of the samples from each group (6 groups for each ceramics, n = 11, 132 in total) were tested, while the other half were subjected to thermocycling (1500 cycles, 5–55 °C, dwell time: 20 s, transition time from one bath to the other: 5 s) prior to testing. Shear bond strength (SBS) was tested using a universal testing machine Z005 (Zwick/Roell) at crosshead speed 2 mm/min, according to ISO/TS 11405 [50].

### 4.3. Statistical Analysis

Statistical analysis of SBS test results was performed. The following statistical parameters were calculated: arithmetic mean (x), median (Me), average values, standard deviation (SD), coefficient of variation (v), and quartile deviation (Qx). Also, minimal and maximal values were given. In order to compare the distribution of shear bond strength values with normal distribution, a Shapiro-Wilk test was performed. As the SBS values in study groups were not normally distributed, non-parametric tests were used to compare SBS mean values. To compare SBS mean values obtained for five different ceramic surface cleaning methods, an ANOVA Kruscal-Wallis (non-parametric equivalent of analysis of variance) test was used. For pairwise comparisons of SBS values obtained in study groups after 24-hour storage and thermocycling, a non-parametric Mann-Whitney test was performed. A level of *p* < 0.05 was considered statistically significant. All the statistical procedures were carried out using STATISTICA 10 (StatSoft, Cracow, Poland).

## 5. Conclusions

The TOF-SIMS analysis showed differences in chemical composition of the saliva-contaminated pre-etched ceramic surfaces after application of different cleaning regimens. However, the differences could not be directly translated into SBS results obtained for tested ceramics. The lowest emission of C_4_H_8_N^+^ ions from the saliva-contaminated ceramic surface might indicate the effectiveness of the surface cleaning method.

In the case of both tested glass ceramics: leucite and lithium disilicate, the shear bond strength (SBS) after artificial ageing—within the statistical significance (*p* < 0.05 and *p* = 0.001, respectively)—depended on the cleaning method used. The most effective cleaning method for the saliva-contaminated LGC surface was cleaning with H_3_PO_4_, while for the LDGC surface it was re-etching with HF. The application of the following cleaning methods resulted in obtaining reliable bond strength.

## Figures and Tables

**Table 1:** Mean shear bond strength values (MPa) ± standard deviation for leucite glass ceramics (Avante) after different cleaning methods. Within cleaning methods, means followed by different letters (uppercase letters in line and lowercase letters in column) indicate significant statistical differences.

Cleaning Method	Storage Conditions	
	24 h	Thermocycling
control	14.59 (±7.63)A	5.46 (±5.24)A
water spray	19.39 (±8.29)Aa	4.70 (±3.93)Aa
ultrasonic bath for 5 min	12.51 (±7.15)A	5.14 (±5.79)A
H_3_PO_4_ for 1 min	12.45 (±9.03)	5.32 (±4.03)b
cleaning paste for 1 min	12.27 (±7.36)A	3.32 (±1.44)Aac
re-etching (9% HF for 20 s)	9.56 (±4.44)Aa	2.08 (±1.48)Aabc

**Table 2:** Mean shear bond strength values (MPa) ± standard deviation for lithium disilicate glass ceramics (IPS e.max) after different cleaning methods. Within cleaning methods, means followed by different letters (uppercase letters in line and lowercase letters in column) indicate significant statistical differences.

Cleaning Method	Storage Conditions	
	24 h	Thermocycling
control	25.72 (±8.11)a	26.98 (±11.97)a
water spray	13.82 (±6.09)ab	19.78 (±9.81)ab
ultrasonic bath for 5 min	8.88 (±4.77)acd	8.76 (±4.49)abcde
H_3_PO_4_ for 1 min	19.63 (±12.80)c	16.92 (±9.74)ac
cleaning paste for 1 min	19.84 (±11.80)d	13.43 (±7.01)ad
re-etching (9% HF for 20 s)	24.59 (±11.60)bd	26.39 (±12.70)e

**Table 3:** Materials used in the study.

Material	Name/Manufacturer	Composition
ceramics	Avanté® Micro Crystal Porcelain/Pentron	SiO_2_, Al_2_O_3_, K_2_O, Na_2_O, CaO, MgO, Li_2_O, F, BaO, ZrO_2_, B_2_O_3_, ZnO, CeO_2_, Y_2_O_3_, TiO_2_, pigments (various metal oxides)
ceramics	IPS e.max Press/Ivoclar Vivadent, Schaan, Liechtenstein	SiO_2_, Li_2_O, K_2_O, MgO, ZnO, Al_2_O_3_, P_2_O_5_ and other oxides
ceramic etchant	Porcelain Etch/Ultradent, Schaan, Liechtenstein	9% buffered hydrofluoric acid
extra-oral cleaning paste for indirect restorations	Ivoclean/Ivoclar Vivadent, Schaan, Liechtenstein	Zirconium oxide 10–15 wt%, Water 65–80 wt%, Polyethylene glycol 8–10 wt%, Sodium hydroxide ≤ 1 wt%, Pigments, additives 4–5 wt%
etching gel	Total Etch/Ivoclar Vivadent, Schaan, Liechtenstein	37% orthophosphoric acid
universal primer	Monobond Plus/Ivoclar Vivadent, Schaan, Liechtenstein	Alcohol solution of silane methacrylate, phosphoric acid methacrylate and sulphide methacrylate
bonding agent	XP Bond/Dentsply, UK	PENTA, TCB, UDMA, TGDMA, HEMA, Nanofiller, Camphorquinone, Stabilizer, Tert-Butanol
flowable composite material	X-flow/Dentsply, UK	Multifunctional acrylate resin, Difunctional methacrylate resin, DGDMA, UV stabilizer, Ethyl-4(dimethylamino)benzoate, Camphorquinone, BHT, Strontium-alumino-sodium-fluoro-phosphor-silicate glass, Highly dispersed silicon dioxide, Iron oxide pigments, Titanium dioxide
condensable composite material	Spectrum TPH®3/ Dentsply, UK	Urethane modified Bis-GMA dimethacrylate resin Ethoxylated Bisphenol A Dimethacrylate 2,2′-Ethylendioxydiethyldimethacrylat
